# Addressing Quality, Safety, and Sustainability Challenges in Artisanal Pico Cheese Production: Proteolysis Indexes, Staphylococci, and Whey Valorization

**DOI:** 10.3390/foods14091487

**Published:** 2025-04-24

**Authors:** Sandra P. A. Câmara, Cristiana Maduro Dias, Hélder P. B. Nunes, Raphael Martin, Francisca Pimentel, Júlia V. Gomes, Maria da Graça A. Silveira, Henrique J. D. Rosa, Airidas Dapkevicius, Alfredo E. S. Borba, Maria de Lurdes N. E. Dapkevicius

**Affiliations:** 1 Institute of Agricultural and Environmental Research and Technology (IITA-A), University of the Azores, Rua Capitão João d’Ávila, Pico da Urze, 9700-042 Angra do Heroísmo, Portugal; camara29@hotmail.com (S.P.A.C.); cristianarodrigues@gmail.com (C.M.D.); helder.pb.nunes@uac.pt (H.P.B.N.); kika_pimentel2000@hotmail.com (F.P.); julia.v.gomes2000@gmail.com (J.V.G.); henrique.jd.rosa@uac.pt (H.J.D.R.); airidas.dapkevicius@uac.pt (A.D.); alfredo.es.borba@uac.pt (A.E.S.B.); 2 Faculty of Agricultural and Environmental Sciences, University of the Azores, 9700-042 Angra do Heroísmo, Portugal; raphael.martin68@hotmail.com (R.M.); maria.ga.silveira@uac.pt (M.d.G.A.S.); 3 Biotechnology Center of Azores (CBA/UAc), University of the Azores, 9700-042 Angra do Heroísmo, Portugal

**Keywords:** artisanal cheese, coagulase-positive *Staphylococcus*, whey cheese, proteolysis, sustainability, safety, circular economy

## Abstract

Artisanal cheeses face unique challenges due to changes in the present approaches to food safety, health, and environmental sustainability. This work aims at tackling such challenges in Pico cheese, by addressing outdated PDO criteria, the need to tackle coagulase-positive staphylococci (CoPS) and to promote circular economy by upgrading cheese whey. Model raw- and pasteurized milk cheeses were prepared with autochthonous lactic acid bacteria (LAB) as inoculants and analyzed for their composition, proteolysis, and microbiological parameters. CoPS were isolated and the risks they pose in terms of One Health evaluated by assessing phenotypic virulence factors and antibiotic resistance patterns. To assess the potential of autochthonous LAB for controlling CoPS, a challenge test was performed. Probiotic *requeijão* was prepared using autochthonous LAB as inoculants for upgrading whey. This work confirmed the need to update Pico cheese specifications regarding proteolysis indexes. Biofilm production was present in all Pico cheese CoPS, but resistance was only found against penicillin and cefoxitin. Adding salt or extending maturation time up to 60 days did not afford the desired level of CoPS control. *Lactococcus lactis* L1C21M1, however, was able to keep CoPS populations at 3 log cfu g^−1^ in the challenge test. *Requeijão* was a suitable substrate for probiotic autochthonous *Lactococcus lactis* L3A21M1 and L3B1M7.

## 1. Introduction

Artisanal cheeses are part of the cultural heritage of many societies, often playing an important role in the economic and environmental sustainability of the regions where they are manufactured. Pico cheese is one of such cultural and economically important products. It is manufactured in Pico Island (Azores archipelago, Portugal), using raw cow’s milk and a traditional, labor-intensive protocol. It has a short maturation period (20 days) at ca. 10 °C, a pH in the vicinity of 5.0, and a high *a*_w_ value (0.943–0.966). The USA FDA classifies a food product as intrinsically safe if its pH value is below 4.2 and its *a*_w_ does not exceed 0.920 [1]. Such conditions would provide a level of protection that is regarded as similar to that afforded by pasteurization [2], but they are not feasible in Pico cheese, making its safety assessment mandatory. This cheese variety was recently characterized from the biochemical and safety point of view, and the main challenges that endanger its survival in the market were identified [3].

Traditional cheeses, as other foods, may serve as a vehicle for the dissemination of certain microorganisms from dairy cows, their environments, and the cheese production environment to humans [4,5]. Staphylococci are among the bacteria of concern in this respect [6]. Coagulase-positive staphylococci (CoPS), such as *Staphylococcus aureus*, can produce enterotoxins in contaminated foods and constitute one of the main causes of foodborne illness worldwide. Their reservoir is the mucosae of animals (including humans and dairy cows), from which we may gain access to milk and dairy products. Their ability to form biofilms promotes their persistence in dairy environments and facilitates their dispersal via cross-contamination [7]. Besides their pathogenic potential for humans, staphylococci from food can act as a reservoir of genetic determinants of virulence [8], and antibiotic resistance [9,10]. CoPS constitute the main hygiene/safety issue in Pico cheese [3]. Physicochemical parameters in Pico cheese are not enough to provide control over this salt-resistant pathogen, although production of staphylococcal enterotoxins has not been detected in cheeses that were not subjected to temperature abuse during transportation [3]. Therefore, as in the case of other artisanal cheeses with short maturation periods, safety assurance can be challenging in Pico cheese, since hardy bacteria, such as CoPS, may persist and become a safety concern [3,11,12,13]. CoPS (e.g., *S. aureus*) are commonly isolated from raw milk, cheesemaking equipment, cheese plant environments, and the hands of manufacturers [14]. The latter reservoir has been considered of special importance in cheeses, such as Pico, that require intensive manipulation during their manufacture [11]. Pico cheese maturation is thought to be mainly led by lactococci, which constitute more than 80% of its microbiota [15]. Although they are much less abundant, lactobacilli and leuconostocs were found to display antimicrobial activity against several cheese borne pathogens [16,17,18]. Autochthonous lactic acid bacteria (LAB) could, thus, provide an additional barrier to control the main bacterial problems in Pico cheese.

Pico cheese specifications set high levels of proteolysis indicators (25 to 34 g/100 g of water-soluble nitrogen, WSN). In spite of having a WSN level that is within the range found in other Iberian raw cow’s milk cheeses [19,20,21], the cheese presently produced in Pico Island does not reach such high levels of proteolysis [3]. The extension and depth of proteolysis in cheese increase with time, so extending maturation time could lead to higher WSN levels. Salt levels also affect proteolysis, by acting upon proteolytic enzymes and on casein [22]. Therefore, it is important to study the impact of production parameters such as salt content and maturation time on proteolysis indexes, to ascertain the feasibility of such criterion.

Whey is an abundant by-product of cheesemaking, with each kilo of cheese resulting in the production of ca. 10 L of whey, a very polluting residue. Additionally, whey-off constitutes an important source of nutrient loss in the dairy production chain, due to its high content in nutritionally valuable protein [23]. Finding ways of upgrading whey would promote the environmental and economical sustainability of the small, family-owned dairies that manufacture Pico cheese, within a circular economy perspective. In Portugal, a whey cheese variety, *requeijão*, is a well-known and appreciated dairy specialty. *Requeijão* manufacture would provide a means of valorizing the whey resulting from Pico cheese, using production technologies that are easy to implement in these small dairies. Some Pico cheese LAB have been demonstrated to possess probiotic properties [18,24,25]. Applying such probiotic strains to the production of *requeijão* would lead to an innovative, high added value product that responds to the health-oriented preferences of the modern consumers.

This work aims at addressing the main challenges that Pico cheese presently faces from a circular technology point of view, by optimizing aspects of its production protocol (maturation time, salt content), using autochthonous LAB to control coagulase-positive staphylococci, and upgrading the resulting whey into probiotic *requeijão*. To better understand the safety challenge posed by CoPS in Pico cheese, the prevalence of virulence factors and antibiotic resistances among CoPS isolates were studied.

## 2. Materials and Methods

### 2.1. Assessing the Effect of Salt Levels and Maturation Time on Physicochemical Characteristics, Maturation Indexes, and Microbiological Profile of Model Cheeses

#### 2.1.1. Experimental Cheese Manufacture

Experimental cheeses were manufactured from raw milk, obtained at the experimental farm of the University of the Azores, following the traditional Pico cheese protocol [26], with 0.2, 0.5, 0.7 and 0.9% NaCl added (*w*/*w*). In short, coagulation of the raw milk was done at 32 °C, in a 10-L vat (FT20 Cheese Vat Armfield, Ringwood, UK). The coagulant used was a commercial rennet (Bio Ren Premium 97P150; 1:150,000; 0.15 g L^−1^ of milk). Coagulation lasted for 1 h, after which the curds were cut in ca. 2.5 cm^3^ pieces and let to whey off for 5 min. Curds were then placed into molds and firmly pressed with the hands of the cheesemaker. Cheeses were salted by applying table salt to each side and were left to drain for 4 h. They were then removed from the molds and left to mature) for 20, 40, or 60 days (10–12 °C; 40–50% RH). During maturation, cheeses were turned every day. Three experimental cheeses, weighing about 250 g (corresponding to 31–28% of artisanal Pico cheese weight) at the end of maturation, from three independent productions, were prepared for each NaCl content and each maturation time.

#### 2.1.2. Physicochemical Analyses

The pH, titratable acidity, NaCl concentration, water activity (*a*_w_), and compositional parameters (dry matter/moisture, protein, fat, and ash) were determined as described by Câmara et al. [3], except for fat, which was assessed using the Gerber method [27].

#### 2.1.3. Proteolysis Parameters

Nitrogen fractions—water-soluble nitrogen (WSN), 12% trichloroacetic acid (TCA)-soluble nitrogen (12% TCAN), and 5% phosphotungstic acid (PTA)-soluble nitrogen (5% PTAN)—were used to assess proteolysis in the matured cheese samples, according to the methodologies described in Macedo and Malcata [28].

#### 2.1.4. Microbiological Analyses

Microbiological analyses of the milk used in cheese manufacture, of the curds, and of the maturated cheese were performed. Total aerobic mesophiles (TAM), LAB, and coagulase-positive staphylococci (CoPS) were enumerated by plating out diluted samples of milk, curd, or cheese onto the appropriated culture media. TAM were counted on Plate Count Agar (Biokar, BK144HA, Allone, France), incubated for 48 h at 30 °C, and CoPS on Baird-Parker Agar (Biokar, BK055HA, Allone, France) with Egg Yolk-Telurite Supplement (Biokar, BS 06008, Allone, France), incubated for 48 h at 37 °C, and LAB on MRS Agar (Biokar, BK089, Allone, France), incubated for 72 h at 30 °C, as described in Câmara et al. [18].

### 2.2. Characterization, Virulence Factors, and Antibiotic Resistance of Coagulase-Positive Isolates Obtained from the Experimental Cheeses

Eleven isolates of coagulase-positive staphylococci, obtained from the Baird–Parker Agar plates used for enumeration, were tested for anaerobic mannitol fermentation, acetoin production (Voges-Proskauer test) [29], gelatin degradation, hemolysis, DNase production [30], and biofilm formation [31]. Their resistance/sensitivity to 22 antibiotics (Table 1) was tested by the Kirby-Bauer method, as described by EUCAST [32] for the antibiotics used in human medicine, and according to CLSI [33]. protocol for the antibiotics used in veterinary medicine. The test medium used was Mueller–Hinton Agar (Merck, 105 435, Darmstadt, Germany), and the antibiotic disks were obtained from OXOID (Basingstoke, UK).

### 2.3. Potential of Autochthonous LAB Cultures for the Control of S. aureus Populations

#### 2.3.1. Effect of an Autochthonous LAB on *S. aureus* Strains (ATCC 9144 or ATCC 25923) in Pasteurized Whey

Whey resulting from the manufacture of one batch of raw milk cheese with no LAB added was pasteurized at 73 °C, for 16 s, and used as substrate to test the growth of *Lactococcus lactis* L1C21M1. For that, 9 × 25 mL of pasteurized whey were inoculated with a 24 h-old culture of *L. lactis* L1C21M1 in MRS broth, at the rate of 1% (*v*/*v*). Three of the tubes thus obtained were incubated without the addition of staphylococcal cultures. In 6 of the tubes, *Staphylococcus aureus* (ATCC 9144 or ATCC 25923) was added to the whey at 1% (*v*/*v*) and kept subsequently at 10 °C (maturation temperature used in Pico cheese manufacture) for 21 days. Each experiment was performed in triplicate. Incubation and sampling times were as described above. At each sampling time (0, 6, 12, 24, and 48 h; 7, 14, and 21 days), LAB counts were performed in MRS Agar, and coagulase-positive staphylococci were enumerated in Baird-Parker Agar, supplemented with Egg Yolk-Tellurite, incubated at 37 °C for 48 h. The obtained log cfu mL^−1^ counts were used to calculate the growth curve parameters—duration of the lag phase (λ), specific growth rate (µ), and maximum populational level attained (A), by means of the Baranyi model, fitted with the help of the DMFit software [34].

#### 2.3.2. Effect of LAB Addition on CoPS Numbers in Raw Milk Cheeses

Experimental cheeses, with autochthonous LAB added, were prepared according to the general protocol described above. The LAB strains used were isolated from traditional, artisanal Pico cheese in previous works and were *Lacticaseibacillus paracasei* L1B1E3, *Leuconostoc pseudomesenteroides* L1C1E6, *L. lactis* L1C21M1 [18], *L. lactis* L3A21M1, and *L. lactis* L3B1M7 [35]. These LAB species have Qualified Presumption of Safety status [36]. L1B1E3, L1C1E6, and L1C21M1 have desirable properties for cheesemaking [18]. L3A21M1 has probiotic properties. Among other desirable traits, it displays β-galactosidase activity. Furthermore, it is a fast-growing, fast-acidifying strain. L1C1E6 grew and acidified the medium well in model broth systems, displayed caseinolytic activity and produced diacetyl [18]. L3A21M1 and L3B1M7 were shown to degrade histamine and cholesterol in model systems [24], and the former is a bacteriocin producer [25]. Prior to cheesemaking, MRS broth cultures of the different LAB strains were used to inoculate (1:1000 *v*/*v*) Skim Milk (OXOID, LP0031, reconstituted as indicated by the manufacturer), which was subsequently incubated at 30 °C for 24 h. Skim milk cultures of the LAB strains were then added to the milk (32 °C) in the coagulation vat (1% *v*/*v*; ca. 10^9^ cfu mL^−1^), and 1 h of incubation was allowed before adding the coagulant. Assessment of pH was performed in samples of milk, curd, and 21-day old cheeses. LAB. All model cheese variations were prepared duplicate (two independent cheese productions).

#### 2.3.3. Assessing the Effect of LAB Addition Level on CoPS Numbers in Pasteurized- and Raw-Milk Cheeses by a Challenge Test

Experimental cheeses were prepared as described above, using either raw or pasteurized (73 °C, 16 s) milk, to which either *L. lactis* L1C21M1 (1% and 2%, *v*/*v*), L3A21M1, L3B1M7 (1% and 2% *v*/*v*), L1C21M1 + L3A21M1 (1% + 1%, *v*/*v*), L1C21M1 + L3B21M1 (1% + 1% *v*/*v*), L3A21M1 + L3B1M7 (1% + 1%, *v*/*v*), or L1C21M1 + L3A21M1 + L3B1M7 (1% + 1% + 1%, *v*/*v*), previously grown in Skim Milk, incubated at 30 °C for 24 h (ca. 10^9^ cfu mL^−1^ at the end of the incubation time) were added. Prior to starting maturation, cheese surfaces were smeared with 200 µL a suspension of *S. aureus* ATCC 9144 in phosphate buffer (10^4^ cfu g^−1^), obtained from an 18 h culture in Nutrient Broth (AES, AZB140802). Maturation lasted 21 days at 10 °C. LAB and CoPS were enumerated as above in samples of raw milk used for cheese manufacture, curds, and 21-day old cheeses. All analyses were performed in duplicate, with each duplicate corresponding to an independent cheese production.

### 2.4. Screening of Whey Cheese as a Vehicle for Lactococcal Strains

#### 2.4.1. Growth of Lactococci in Whey

Whey was pasteurized and inoculated with the three lactococcal strains under study (*L. lactis* L1C21M1, L3A21M1, and L3B1M7), as described above. Two independent whey cultures of each strain were then incubated at 4 °C for 21 days. LAB counts were performed at 0, 6, 12, 24, 28, 168, 336, and 504 h, as described above. The obtained log cfu mL^−1^ counts were used to calculate the growth curve parameters—duration of the lag phase (λ) and specific growth rate (µ)—by means of the Baranyi model, fitted with the help of the DMFit software [34].

#### 2.4.2. Fate of Autochthonous Lactococci in *requeijão*

Whey cheese (*requeijão*) was prepared by pasteurizing the whey that resulted from the experimental cheese protocol at 73 °C, for 16 s. Subsequently, the whey was heated to 85 °C, to perform the acid coagulation step. The coagulation of the whey protein was then achieved by adding 1% commercial wine vinegar. After cooling, the mixture was strained through a cheesecloth, 1% (*w*/*w*) of table salt was added, the salted curd mass was placed into molds and stored under at 4 °C for a maximum of 7 days. *L. lactis* L1C21M1 (2%, *v*/*w*) or a combination of *L. lactis* L1C21M1 and L3A21M1 (1% each, *v*/*w*) were added to the curds at the salting step.

Each whey cheese variety was sampled at day 0 and at day 7 of storage, and the pH and LAB counts of the resulting samples were determined as described above. All analyses were performed in duplicate.

### 2.5. Statistical Analyses

The effects of the level of NaCl addition and the cheese manufacture stage upon the various physical, chemical, and microbiological parameters were tested by factorial Analysis of Variance (ANOVA). Whenever ANOVA detected significant differences within these two factors, multiple comparisons were performed using Tukey’s test. All tests were performed using the SPSS Software Package 25, v. 30.0.0.0 (IBM Corporation, New York, NY, USA).

## 3. Results

### 3.1. Effects of Maturation Time and Salt Addition Levels on Physicochemical Parameters, Proteolysis Indexes, and Microbial Populations in Experimental Cheeses

Table 2 shows the results of compositional analyses of the experimental cheeses, prepared from raw milk, to assess the effect of maturation length and salt addition level. The raw milk used had a protein content of 3.90 ± 0.41 and a fat content of 4.49 ± 0.22. Moisture decreased significantly (*p* < 0.05) as maturation time progressed but was not significantly (*p* > 0.05) affected by salt addition level. In 20-day-old cheeses, moisture averaged 42.1 ± 0.5 g 100g^−1^ of cheese, reaching 28.4 ± 0.5 at 60 days. Neither maturation time nor salt addition level affected significantly (*p* > 0.05) the observed ash and protein levels, which remained at 5.6 ± 0.1 and 38.8 ± 0.4 g 100 g^−1^ of TS, respectively. Small, but significant (*p* < 0.05) differences were found in fat content between 20-day old cheeses (48.2 g 100 g^−1^ of TS, on average) and their counterparts that had aged for 40 and 60 days (50.3 and 51.1 g 100 g^−1^ of TS, in average, respectively). Salt addition level did not significantly (*p* > 0.05) affect the fat content of the experimental cheeses. Predictably, salt addition level significantly (*p* < 0.05) affected the NaCl content of the cheeses, and so did the length of maturation. In cheeses that received higher amounts of salt, NaCl content increased faster than in those receiving lower amounts. At the end of maturation, NaCl in cheese TS ranged from 0.4 to 1.6 g 100 g^−1^.

Maturation indexes (WNS, 12%TCAN, and 5%PTAN, on a TN basis) were not significantly (*p* > 0.05) affected by salt addition at the studied levels. However, 20 days old cheeses differed significantly (*p* < 0.05) from the 40- and 60-day-old ones in their WSN and 12% TCAN values. WSN values decreased as the maturation time increased. Cheese WSN averaged 13.6, 9.3, and 8.2 g 100 g^−1^ of TN at, respectively, 20, 40, and 60 days of maturation. The same trend was observed for 12% TCAN, which averaged 8.6, 5.5, and 4.6 g 100 g^−1^ of TN at 20, 40, and 60 days, respectively. No significant (*p* > 0.05) changes were observed in the 5% PTAN values as maturation time increased. The average 5% PTAN value of cheeses was 1.24 g 100 g^−1^ of TN.

Table 3 shows the effect of manufacture stages and salt addition levels on pH, titratable acidity, *a*_w_, salt-in-moisture, TAM, LAB, and CoPS population counts. Differences in the pH value of the raw milk used in the manufacture of the experimental cheeses, and in the curds were not considerable, with pH values of about 6.7 to 6.9 in the former and 6.7 to 6.8 in latter. A significant (*p* < 0.05) decrease in pH, exceeding 1 unit, was observed from curd to the 20-day-old cheeses. Further significant (*p* < 0.05) decreases in pH were observed between 40 and 60 days of maturation. At the end of the 60-day maturation period, cheeses had a pH value of about 5. Differences in the pH of cheeses manufactured with different levels of added salt were small, albeit statistically significant (*p* < 0.05). The titratable acidity values of the experimental cheeses increased significantly (*p* < 0.05) from 20 to 40 days of maturation. Significant (*p* < 0.05) differences were also observed between cheeses with 0.5% and 0.7% added salt. Titratable acidity levels at the beginning of maturation ranged from 0.8% to 1.2%. After 60 days, the titratable acidity of the experimental cheeses varied from 1.5% to 2.3%. When considering titratable acidity, it should be kept in mind that, in cheese, this parameter is more a measure of the buffering capacity than of the amount of acid accumulated during lactose fermentation [37]. In the experimental cheeses, *a*_w_ values were always below or close to the 0.6 limit for microbial growth, and markedly lower than those observed in Pico cheese curds (ca. 0.97) [3]. Differences were significant (*p* < 0.05) between 20 days and both other tested maturation periods, as well as for the 0.2%, 0.5%, and 0.7% salt addition levels. As expected, salt-in-moisture values increased significantly (*p* < 0.05) with increasing levels of salt addition. They also increased significantly (*p* < 0.05) from 30 to 40 days of maturation. Values of salt-in-moisture ranged from 1.0–5.6 g 100 g^−1^.

This work showed that duplicating or triplicating the length of maturation did not result in an increase in the proteolysis indexes. In the later stages of maturation, proteolysis derives from the action of starter and non-starter LAB. In our model cheeses, as well as in Pico cheese, LAB populations remain high throughout the maturation period. In this study, LAB populations remained practically unchanged even after 60 days of maturation. LAB autolysis is important to release the peptidases that will breakdown the peptides released under the proteolytic action of rennet during the first stages of maturation. Autochthonous Pico cheese LAB have previously been shown to promote proteolysis, a central event in maturation that is implicated in cheese texture and flavor development [26]. Hence, the high numbers of LAB may help explaining why an increase in proteolysis indexes was not observed. Another source of proteolytic activity is raw milk’s endogenous enzymes. Several factors may affect this activity, some of which, such as the somatic cell counts, are impacted by dairy farm management [38]. Pico cheese specifications were published almost three decades ago, and many aspects of milk production, among which somatic cell count (SCC) status in the Azorean dairy effective, have changed. For instance, in SCC values in the milk used in our study were always below 2 × 10^4^ (data obtained from the farm’s records). Thus, the high proteolysis indexes in the specifications may no longer reflect the present reality—nor do they necessarily reflect the preferences of today’s consumers. They should, therefore, be revised.

No significant (*p* > 0.05) effect of salt addition was observed on the counts of TAM, LAB, and enterobacteria. Changes in TAM numbers form milk to curd were of 0.1–0.4 log cycles. TAM populations reached population levels close to 4 log cfu g^−1^ in the latter. Population levels in 20-day-old cheese were close to 9 log cfu g^−1^, representing a considerable, significant (*p* < 0.05) increase. A subsequent decrease of ca. 1 log cycle in TAM was observed, leading to populations that were close to 9 log cfu g^−1^. Differences in TAM populations between raw milk, curd, and the matured cheeses were significant (*p* < 0.05). They were also significant (*p* < 0.05) between cheeses at 60 days of maturation and those that had been matured for 20 or 40 days.

Raw milk, curd, and the matured cheeses differed significantly (*p* < 0.05) in their LAB population sizes. In the curds, LAB were less numerous than in the raw milk and were close to the detection limit of the enumeration method used. A 7–9 log cycle increase in LAB populations was observed from curd to cheese, with populations close to 9 log cfu g^−1^ in the latter. LAB populations remained stable throughout the rest of the maturation period (days 20 to 60). As Gram-positive bacteria, LAB are well adapted to osmolality challenges, making them able to survive and persist in relatively low aw environments [39], such as those provided by maturing cheeses, which explains their high numbers in Pico cheese. As Table 3 shows, LAB numbers do not increase from days 20 to 60 of maturation, indicating survival rather than growth during this period, when aw values have fallen below the minimal value that supports their multiplication. The increase in LAB numbers from curd to 20-day old cheeses would have taken place in the curd and/or during the very early stages of maturation, when moisture (Table 2) and aw values were high enough for their growth, in accordance with reports by Medved’ová et al. [40] and Settanni and Moschetti [41] that LAB growth occurs largely in the first two weeks of maturation.

CoPS populations in the milk were 2–3 log cfu mL^−1^. An increase of about 3–4 log cycles was observed from the curds to the 20-day-old cheeses, with a subsequent decrease. Maximum populations were in the range of 4–6 log cfu g^−1^ cheese. Differences between cheeses maturated for 20, 40, and 60 days were not significant (*p* < 0.05). CoPS populations were higher in the cheeses manufactured with higher (0.7 and 0.9%) levels of salt addition than in those that had less added salt (0.2 and 0.5%). Differences between CoPS numbers in the cheeses with 0.2 to 0.7% added salt were significant (*p* < 0.05). Thus, higher levels of NaCl addition seemed to favor these salt-tolerant microorganisms.

### 3.2. Characterization, Virulence Factors and Antibiotic Resistance of Coagulase-Positive Isolates Obtained from the Experimental Cheeses

All CoPS isolated from the experimental cheeses were able to ferment mannitol under anaerobic conditions and produced acetoin, indicating that they possibly belong to the *S. aureus* species [29]. Results of the tests for virulence factors and antibiotic resistance are shown in Table 4. None of the tested CoPS were able to hydrolyze gelatin, but most were DNAse positive. All were α-hemolytic and produced biofilm to different degrees under the employed test conditions. Gelatinase production is an important virulence factor in Gram-positive cocci. It hydrolyzes collagen and is associated with the ability to invade the host [42]. DNAse is highly immunogenic and is involved in escaping neutrophile extracellular traps in other Gram-positive pathogens [43]. Biofilm production was very common among the tested isolates. Most of them could be classified as strong biofilm producers at 48 h, according to the criteria used by Ribeiro et al. [44]. Biofilm production is a well-known virulence factor among staphylococci. It confers increased resistance both to the host’s clearance mechanisms and to treatment with antimicrobial agents, promoting dissemination of the pathogen within the host, and leading to chronic infections [45]. Biofilm formation also makes staphylococci hard to eradicate from milk contact surfaces by the commonly used cleaning and sanitation programs, and promotes their persistence in milk-associated environments, increasing the chances for cross-contamination [46] and making them especially hard to control in these environments.

Resistance was observed against only two of the tested antibiotics: penicillin and cefoxitin, both of which are β-lactams. Resistance to penicillins is linked to β-lactamase production, encoded in the *blaZ* gene, located either on plasmids or on the bacterial chromosome. Frequent carriage of *blaZ* and, consequently, penicillinase production, with the concomitant phenotypic resistance to penicillins, has been described among cheese staphylococci [47], a situation that we did not find in our isolates, since only two were resistant to penicillin. Cefoxitin resistance is mediated by the production of a penicillin-binding protein that has low affinity for β-lactams (PBP2), encoded by *mecA*, which is part of a chromosomal genomic island [48]. Since distinct resistance mechanisms are involved, staphylococci that are resistant to penicillins may still be sensitive to penicillinase-resistant β-lactams, such as cephalosporins. Most of the isolates from the experimental cheeses, except for those that were resistant to penicillin, displayed resistant phenotypes to cefoxitin. In *S. aureus*, the cefoxitin disk assay is used to detect methicillin resistance [33]. Methicillin-resistant *S. aureus* (MRSA) are important both as animal, and as human pathogens. Their presence in raw-milk cheeses has been previously documented [49], and these foods may have a role both in the dissemination of MRSA strains, and of their genetic determinants of resistance, making their control important in a One Health perspective.

### 3.3. Screening of Whey Cheese as a Vehicle for Lactococcal Strains

#### 3.3.1. Effect of Autochthonous LAB on the Growth of *S. aureus* Strains in a Whey Model

To assess the potential of autochthonous LAB for the control of *S. aureus*, *L. lactis* L1C21M1 and two reference strains of the former (ATCC 9144 and ATCC 25923—a methicillin-resistant strain) were cultured in a model system, consisting of pasteurized cheese whey (no LAB or CoPS detected), and incubated for 21 days at 10 °C, to simulate the maturation time of artisanal Pico cheese. As Table 5 shows, the growth of both *S. aureus* strains was hindered in the presence of the autochthonous *L. lactis* L1C21M1, resulting in staphylococcal populations that were, by the end of the incubation time, a log cycle lower than when staphylococci were grown alone in whey. These results highlight the potential of autochthonous lactococci to keep staphylococcal populations close to their initial levels in dairy model systems. However, it must be noted that the effect of the studied autochthonous *Lactococcus* strain upon the two staphylococcal strains used was bacteriostatic, rather than bactericidal.

#### 3.3.2. Effect of Adding Autochthonous LAB to Raw-Milk Model Cheeses

**Figure 1:**
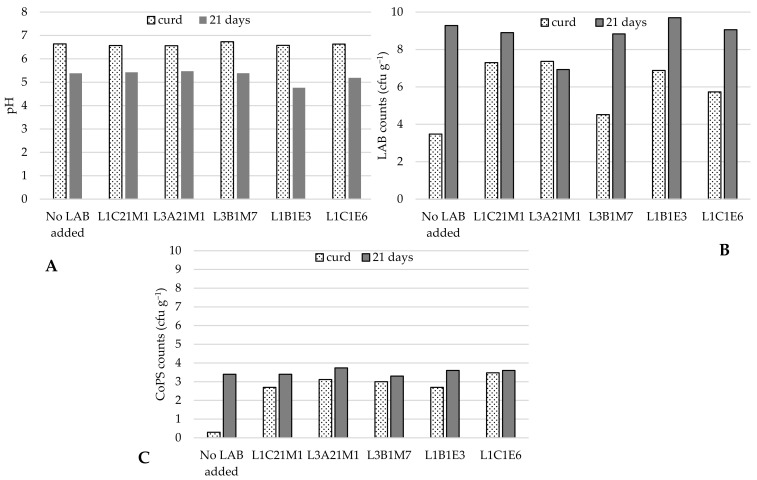
pH (**A**), LAB (**B**), and CoPS (**C**) populations in curd and 21-day old experimental cheeses made from raw-milk. LAB—lactic acid bacteria; CoPS—coagulase-positive staphylococci. The bars represent average values (*n* = 2).

Five autochthonous LAB, of which three were lactococci (*L. lactis* L1C21M1, L3A21M1, and L1C21M1), were used to prepare raw-milk model cheeses. As Figure 1 shows, when no autochthonous LAB were added, CoPS populations increased by 3 log cycles in 21-day old cheeses in comparison with the respective curds. LAB addition kept CoPS populations stable and under log 4 cfu g^−1^, similarly to what Wörmann et al. [49] found. However, *Lb. paracasei* L1B1E3 led to an excessively low pH in cheese, which is not characteristic of Pico cheese, and therefore yielded cheeses with an undesirable texture. These results are in line with those of previous experiments, in which it was demonstrated that this strain has a high acidification capacity [18]. Cheeses made with the addition of *Ln*. *pseudomesenteroides* L1C1E6 also did not have a desirable texture, and developed an atypical slimy crust, which is not desirable in Pico cheese. Therefore, only the lactococcal strains were used in further tests.

#### 3.3.3. Effect of Adding Autochthonous Lactococci on CoPS Levels in Pasteurized and Raw Milk Cheese Models by a Challenge Test

Given the potential of the autochthonous lactococci to control staphylococcal populations in the whey system, model cheeses were prepared from raw or pasteurized milk inoculated with three autochthonous lactococcal strains (L1C21M1, L3A21M1, and L3B1M7), added at 1% and 2% (*v*/*v*) to the milk prior to coagulation. Combinations of these strains (1% + 1%, *v*/*v*) were also used as inoculants. The cheese surface was contaminated with *S. aureus* ATCC 9144, to simulate post-manufacture contamination/cross contamination. In previous studies [17], L1C21M1 provided good curd consistency, lower whey release from the curds, demonstrated activity of several enzymes with beneficial impact on flavor, and produced diacetyl, a compound linked to buttery flavor in dairy products, a desirable flavor note in Pico cheese. L3A21M1 has β-galactosidase activity, produces the bacteriocin lacticin 481 [25] and can survive gastrointestinal tract conditions when included in a cheese matrix [50], whereas L3B1M7 belongs to a cluster of LAB expressing desirable enzymatic activities for cheese production (esterases, peptidases, acid phosphatase, and phosphohydrolase) [51]. All tested isolates were devoid of relevant undesirable properties [18,50,51] and belong to QPS species [36].

**Figure 2:**
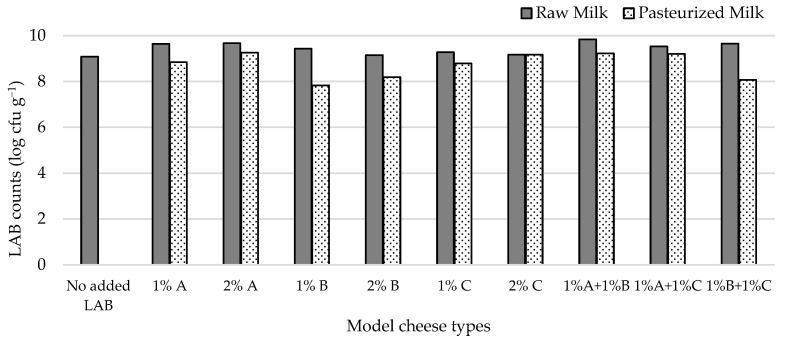
LAB populations in 21-day-old experimental cheeses made from raw (RM) and pasteurized (PM) milk with added *L. lactis* L3A21M1 (A), L3B1M7 (B), and/or L1C21M1 (C), at 1% or 2% (*v*/*v*), surface-contaminated with *S. aureus* ATCC 9144.

Figure 2 shows that, under the conditions we tested, LAB attained populations in the model cheeses that ranged from 7.8–9.8 log cfu g^−1^. All inoculated cheese types had higher LAB counts than the non-inoculated one, the highest difference pertaining to cheese made from milk inoculated with 1% L2A21M1 + 1% L3B1M7. Adding 2% of the studied strains to milk prior to cheese manufacture did not lead to expressive increases in LAB counts when compared with adding 1% of inoculant. LAB numbers in raw-milk cheeses were always higher than in their pasteurized milk counterparts, except for those that contained L1C21M1, which had identical LAB populations in both inoculation levels. The higher LAB numbers in raw milk cheeses may reflect the presence of non-starter LAB (NSLAB). The similar performance of L1C21M1 in raw- and pasteurized-milk cheeses might, thus, reflect its ability to compete with NSLAB.

**Figure 3:**
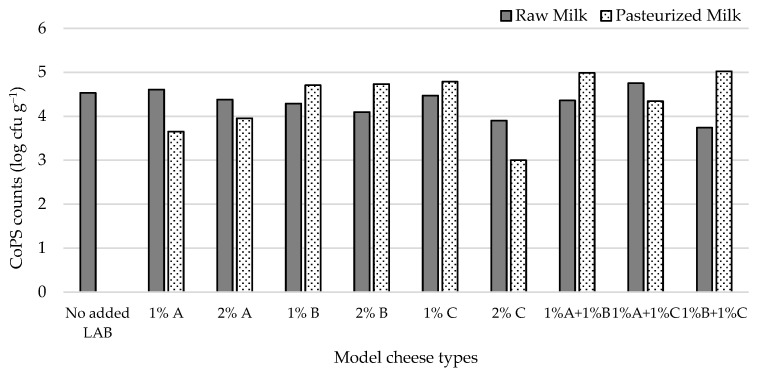
CoPS populations in 21-day old experimental cheeses made from raw (RM) and pasteurized (PM) milk with added *L. lactis*, L3A21M1 (A), L3B1M7 (B) and/or L1C21M1 (C), at 1% or 2% (*v*/*v*), surface-contaminated with *S. aureus* ATCC 9144.

CoPS numbers in raw milk cheeses (Figure 3) ranged from 3.7 (1% L3B1M7 + 1% L1C21M1) to 4.8 log cfu g^−1^ (1% L3A21M1 + 1% L1C21M1). Inoculating the cheese surface with *S. aureus* ATCC 9144 resulted in high CoPS populations in the cheese mass, showing that these bacteria can migrate from the cheese surface towards its interior. The obtained CoPS numbers were within the range of those found in commercial Pico cheese [3]. Most inoculants tested resulted in raw milk cheeses with lower CoPS numbers than the non-inoculated control (4.5 log cfu g^−1^). In pasteurized milk cheeses, CoPS counts ranged from 3.00 (2% L1C21M1) to 5.02 log cfu g^−1^ (1% L3A21M1 + 1% L3B1M7). When co-inoculated with *S. aureus* in pasteurized milk, the strains under study afforded different levels of control of the pathogen. L1C21M1 led, at the 2% inoculation level, to CoPS populations that were two log cycles lower than the 5 cfu g^−1^ threshold for enterotoxin production [52], while for L3A21M1 (at both inoculation levels) CoPS counts were only one log cycle below that limit. These three types of inoculants performed better in the pasteurized milk cheese model than they had in the raw milk cheese. None of the other inoculant types were able to control CoPS populations in the tested pasteurized cheese model, with most of them performing worse than they had in the raw milk model. These results may indicate that L1C21M1 and L3A21M1 can effectively compete with staphylococci in our cheese model. Contrarily to what was observed in our cheese model, MRS broth cultures of L1C21M1 did not inhibit *S. aureus* ATCC 9144 when tested by an agar diffusion assay [18], highlighting the need to use model systems that are as close to reality as possible when assessing LAB strains for their antimicrobial activity. However, this strain was able to initiate grow rapidly and had a high growth rate [18]. Its growth potential might be favorable in the challenging cheese environment, providing it with competitive advantage over staphylococci. L3A21M1 produces lacticin 481, a tricyclic lantibiotic bacteriocin with a broad antibacterial spectrum against Gram-positive bacteria [25]. Its activity against *S. aureus* has not been described, however.

Although controlling *S. aureus* in Pico-style experimental cheeses remains challenging, L1C21M1, added at 2% (*v*/*v*) to milk prior to coagulation (ca. 2 log cfu mL^−1^) demonstrated ability to keep this pathogen at counts well below the 5 log cfu g^−1^ limit for enterotoxin production. We have previously demonstrated that, even when coagulase-positive staphylococcal populations reach or slightly surpass the 5 log cfu g^−1^, enterotoxin production does not occur under Pico cheese maturation conditions [3]. However, it is still important to keep CoPS populations low, to address their potential dissemination throughout the food chain and their possible role as reservoirs of methicillin resistance, under the One Health approach. As such, refrigeration during distribution, sale, and at the consumer level should be scrupulously applied.

### 3.4. Screening of Whey Cheese as a Vehicle for Lactococcal Strains with Probiotic Potential

#### 3.4.1. Fate of Autochthonous Lactococci in Whey

The three lactococcal strains under study displayed different behaviors when incubated in whey, under refrigeration (4 °C), representing the storage conditions of Portuguese whey cheese (*requeijão*). As shown in Table 6, L1C21M1 and L3B1M7 grew, respectively, 1 and 3 log cycles in 21 days, while L3A21M1 survived, in spite of a slight decrease (Table 6). L1C21M1 and L3B1M7 had similar maximum specific growth rates, although the latter had a longer lag phase. This shows that the lactococcal strains under study are well adapted to whey and can survive or even grow during refrigerated storage in this substrate, making them promising for the development of whey-based products.

#### 3.4.2. Fate of Autochthonous Lactococci in Whey Cheese

**Figure 4:**
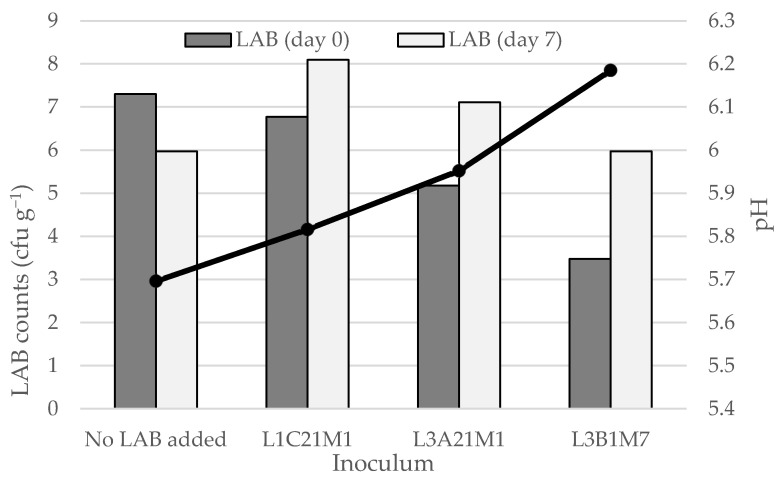
LAB counts and pH decrease in *requeijão* prepared from the whey obtained during the manufacture of model Pico cheeses with or without adding three lactococcal strains (L1C21M1, L3A21M1, and L3B1M7). LAB—lactic acid bacteria. The bars represent average values (*n* = 2).

As shown in Figure 4, whey cheese (*requeijão*) stored at 4 °C contained LAB populations at 6–8 log cfu g^−1^, even at the end of its intended shelf life (7 days). In the non-inoculated whey cheeses and in those that were inoculated with *L. casei* L1C21M1, LAB counts at the end of the storage period barely reached the 6 log cfu g^−1^. In the latter, LAB counts decreased by more than one log cycle. L1C21M1 demonstrated a high autolytic rate in previous studies [3], and that could explain its decrease in *requeijão*. On the opposite, LAB counts increased in the *requeijões* that were inoculated with L3A21M1 and L3B1M7, reaching population densities of 8.1 and 7.1, respectively. These population densities are within the range that is needed for *requeijão* containing L3A21M1 and L3B1M7 to be regarded a probiotic food [53]. The bacteriocinogenic properties of these strains, in particular of the former [35], may account for their ability to yield higher populations, by eliminating potential competitor wild LAB present in the *requeijão*. The pH decrease was deeper in inoculated *requeijões* than in the non-inoculated control. However, the observed pH decrease was not pronounced, ranging from 0.3 (control) to 0.8 (inoculated with L1C21M1). This is an important aspect, since *requeijão* is appreciated by its consumers for its mild taste. Our results indicate that *requeijão* appears to be a promising carrier for probiotic LAB. Strain L3A21M1 seems particularly suited for this purpose, due to the high populations it reached, coupled with the minimal changes in pH it yielded. This strain has β-galactosidase activity, that may contribute, in the human gut, to alleviate lactose intolerance, and leads to the accumulation of prebiotic, bifidogenic galactooligosaccharides. It degrades histamine and cholesterol and has demonstrated in vitro antimicrobial activity against foodborne pathogens [18,24,25].

## 4. Conclusions

This work suggests that the studied autochthonous LAB are useful technological tools, both in terms of flavor and texture development in Pico cheese, and in terms of safety since they promote proteolysis and may constitute an additional hurdle in the control of the most relevant hygiene concern (CoPS).

Neither salt addition nor prolonging maturation time had a significant effect on proteolysis indexes. Although using autochthonous LAB may be beneficial in that respect, revision of Pico cheese specifications should be performed, to approximate WSN target values to those that are feasible in the present production conditions.

Our findings demonstrate that controlling CoPS in Pico cheese remains challenging. Higher levels of salt addition did not prove useful and might even select for staphylococci against LAB. Prolonged incubation times did not bring considerable reductions in CoPS populations in cheeses made from raw milk, either. However, adding autochthonous LAB did afford some control over this pathogen in the cheeses, keeping its populations well below the generally acknowledged limit for enterotoxin accumulation, even when challenged with contamination of the cheese surface with *Staph aureus*. LAB addition levels should be the object of deeper investigation before considering their application in Pico cheese manufacture. LAB addition should not, however, replace other hurdles against CoPS growth, such as control of their access to milk (milking hygiene, mastitis control), and applying refrigeration throughout Pico cheese’s shelf life.

The whey resulting from Pico cheese production can be valorized into probiotic *requeijão*, promoting circular technology practices, and maximizing the usage of dairy resources, while contributing to mitigate the polluting load related to cheesemaking effluents. *Requeijão* provided a good substrate for the tested LAB strains, which were present in sufficiently high numbers to provide probiotic activity.

## Figures and Tables

**Table 1:** List of the antibiotics used for testing resistance/sensitivity of 12 coagulase-positive staphylococci isolated from experimental cheeses.

Antibiotic Target	Classification	Antibiotic(s)	Disk Charge (µg) ^1^
Cell wall	Penicillins	Penicillin G	1 unit
	Cephalosporins	Cefoxitin ^2^	30
		Cefoperazone ^3^	30
		Ceftiofur ^3^	30
		Ceftaroline ^4^	30
Protein synthesis	Aminoglycosides	Gentamycin	10
		Kanamycin	30
		Tobramycin	30
	Tetracyclines	Tetracycline	30
		Minocycline	30
	Phenicols	Chloramphenicol	30
	Fusidanes	Fusidic acid	50
	Macrolides	Erythromycin	15
	Ansamycins	Rifampicin	5
	Oxazolidinones	Linezolid	10
	Streptogramins	Quinupristin-dalfopristin	15
	Monocarboxylic acids	Mupirocin	200
Folate synthesis	Diaminopyrimidines	Trimethoprim	5
	Sulfonamide-diaminopyrimidine	Sulfamethoxazole-trimethoprim	25
DNA	Fluoroquinolones	Moxifloxacin	5
		Norfloxacin	10
Cell wall—DNA	Penicillin-aminocoumarin	Penicillin-novobiocin	40

^1^ Unless otherwise stated. ^2^ 2nd generation; ^3^ 3rd generation; ^4^ 5th generation.

**Table 2:** Compositional parameters and proteolysis indexes of experimental Pico-style cheeses made from raw milk with different levels of salt addition (0.2, 0.5, 0.7, and 0.9%) at different maturation times (20, 40, and 60 days). WSN—water soluble nitrogen; TCAN—tricholoracetic acetic acid-extractable nitrogen; phosphotungstic acid-extractable nitrogen. Values are the average ± standard deviation (*n* = 3). Salt addition levels and maturation days with different superscripts differ significantly (*p* < 0.05).

Parameters	Maturation Time (d)	Added Salt (g 100^−1^ Cheese)			
**Moisture** **(g 100 g** ^−**1**^ **of cheese)**		**0.2** ^a^	**0.5** ^a^	**0.7** ^a^	**0.9** ^a^
	**20** ^a^	42.0 ± 1.8	41.3 ± 2.1	42.5 ± 1.4	42.5 ± 1.5
	**40** ^b^	31.5 ± 0.9	33.7 ± 2.1	32.2 ± 1.6	32.4 ± 3.1
	**60** ^c^	29.5 ± 0.1	28.2 ± 1.9	28.2 ± 1.3	28.0 ± 1.9
**Ash** **(g 100 g** ^−**1**^ **TS)**		**0.2** ^a^	**0.5** ^a^	**0.7** ^ab^	**0.9** ^b^
	**20** ^a^	5.1 ± 04	5.2 ± 0.4	5.6 ± 0.3	6.2 ± 0.3
	**40** ^a^	5.9 ± 1.6	5.2 ± 0.0	5.8 ± 0.3	6.2 ± 0.3
	**60** ^a^	5.2 ± 0.1	5.5 ± 0.2	5.4 ± 0.3	6.0 ± 0.0
**Protein** **(g 100 g** ^−**1**^ **TS)**		**0.2** ^a^	**0.5** ^a^	**0.7** ^a^	**0.9** ^a^
	**20** ^a^	39.8 ± 0.3	39.4 ± 2.0	36.4 ± 5.7	38.6 ± 1.9
	**40** ^a^	39.7 ± 0.1	39.4 ± 0.8	39.5 ± 0.3	38.3 ± 1.1
	**60** ^a^	39.4 ± 0.3	38.9 ± 1.3	37.1 ± 4.2	38.8 ± 1.8
**Fat** **(g 100 ^g−1^ TS)**		**0.2** ^a^	**0.5** ^a^	**0.7** ^a^	**0.9** ^a^
	**20** ^a^	48.5 ± 0.2	48.2 ± 0.7	47.5 ± 1.1	48.4 ± 1.4
	**40** ^b^	48.6 ± 2.4	51.3 ± 1.6	50.6 ± 2.0	50.8 ± 1.5
	**60** ^b^	50.5 ± 0.8	52.0 ± 1.7	51.9 ± 0.3	49.9 ± 1.3
**NaCl** **(g 100 g** ^−**1**^ **TS)**		**0.2** ^a^	**0.5** ^b^	**0.7** ^c^	**0.9** ^d^
	**20** ^a^	0.4 ± 0.0	0.7 ± 0.0	1.0 ± 0.1	1.6 ± 0.1
	**40** ^b^	0.5 ± 0.0	0.8 ± 0.0	1.2 ± 0.1	1.6 ± 0.1
	**60** ^c^	0.5 ± 0.0	0.9 ± 0.1	1.4 ± 0.1	1.6 ± 0.1
**WSN** **(g 100 g** ^−**1**^ **TN)**		**0.2** ^a^	**0.5** ^a^	**0.7** ^a^	**0.9** ^a^
	**20** ^a^	11.2 ± 2.7	11.8 ± 5.1	17.5 ± 7.0	13.9 ± 4.4
	**40** ^b^	10.5 ± 2.7	8.3 ± 0.6	9.1 ± 1.5	9.3 ± 1.2
	**60** ^b^	7.9 ± 0.9	7.9 ± 0.5	8.7 ± 2.1	8.4 ± 1.3
**12% TCAN** **(g 100 g** ^−**1**^ **TN)**		**0.2** ^a^	**0.5** ^a^	**0.7** ^a^	**0.9** ^a^
	**20** ^a^	8.4 ± 1.7	6.5 ± 3.5	10.2 ± 3.7	9.3 ± 3.1
	**40** ^b^	6.3 ± 1.0	4.7 ± 1.1	5.1 ± 1.4	5.9 ± 1.8
	**60** ^b^	5.4 ± 0.3	3.7 ± 0.7	5.8 ± 1.0	3.5 ± 0.5
**5% PTAN** **(g 100 g** ^−**1**^ **TN)**		**0.2** ^a^	**0.5** ^a^	**0.7** ^a^	**0.9** ^a^
	**20** ^a^	0.4 ± 0.0	0.7 ± 0.0	1.0 ± 0.1	1.6 ± 0.1
	**40** ^a^	0.5 ± 0.0	0.8 ± 0.0	1.2 ± 0.1	1.6 ± 0.1
	**60** ^a^	0.5 ± 0.0	0.9 ± 0.1	1.4 ± 0.1	1.6 ± 0.1

**Table 3:** pH, titratable acidity, *a*_w_, and microbial counts of experimental cheeses with different levels of salt addition (0.2, 0.5, 0.7, and 0.9%) at different manufacture stages (raw milk, curd, cheeses at 20, 40, and 60 days of maturation). TAM—total aerobic mesophiles; LAB—lactic acid bacteria; CoPS—coagulase-positive staphylococci. Values are the average ± standard deviation (*n* = 3). Salt addition levels and manufacture stages with different superscripts differ significantly (*p* < 0.05).

Parameters	Manufacture Stage	Added Salt (g ^100−1^ Cheese)			
**pH**		**0.2** ^a^	**0.5** ^ab^	**0.7** ^a^	**0.9** ^b^
	**Raw milk** ^a^	6.86 ± 0.05	6.86 ± 0.01	6.73 ± 0.01	6.82 ± 0.01
	**Curd** ^a^	6.77 ± 0.05	6.72 ± 0.02	6.74 ± 0.02	6.74 ± 0.02
	**Cheese, 20 days** ^b^	5.39 ± 0.25	5.51 ± 0.11	5.67 ± 0.02	5.67 ± 0.16
	**Cheese, 40 days** ^c^	5.18 ± 0.23	5.41 ± 0.08	5.15 ± 0.14	5.55 ± 0.10
	**Cheese, 60 days** ^d^	5.08 ± 0.12	5.20 ± 0.08	5.09 ± 0.14	5.41 ± 0.11
**Titratable acidity**		**0.2** ^ab^	**0.5** ^a^	**0.7** ^b^	**0.9** ^ab^
	**Cheese, 20 days** ^a^	1.18 ± 0.42	1.05 ± 0.04	0.86 ± 0.12	0.77 ± 0.14
	**Cheese, 40 days** ^b^	1.31 ± 0.06	1.07 ± 0.06	1.97 ± 0.30	1.74 ± 0.30
	**Cheese, 60 days** ^b^	1.76 ± 0.00	1.30 ± 0.11	2.27 ± 0.42	1.84 ± 0.31
***a*_w_**		**0.2** ^a^	**0.5** ^b^	**0.7** ^c^	**0.9** ^a^
	**Cheese, 20 days** ^a^	0.63 ± 0.02	0.80 ± 0.01	0.74 ± 0.01	0.64 ± 0.01
	**Cheese, 40 days** ^b^	0.58 ± 0.00	0.62 ± 0.00	0.62 ± 0.02	0.59 ± 0.01
	**Cheese, 60 days** ^b^	0.63 ± 0.00	0.58 ± 0.00	0.59 ± 0.00	0.62 ± 0.00
**Salt-in-moisture**		**0.2** ^a^	**0.5** ^b^	**0.7** ^c^	**0.9** ^d^
	**Cheese, 20 days** ^a^	1.00 ± 0.04	1.69 ± 0.08	3.36 ± 0.42	2.10 ± 0.93
	**Cheese, 40 days** ^b^	1.46 ± 0.09	2.42 ± 0.18	4.94 ± 0.67	3.22 ± 1.47
	**Cheese, 60 days** ^c^	1.76 ± 0.00	3.14 ± 0.51	5.63 ± 0.24	3.66 ± 1.51
**TAM counts (log cfu g** ^−**1**^ **)**		**0.2** ^a^	**0.5** ^a^	**0.7** ^a^	**0.9** ^a^
	**Raw milk** ^a^	3.52 ± 0.28	4.12 ± 0.42	3.41 ± 0.17	3.47 ± 0.15
	**Curd** ^b^	3.67 ± 0.49	4.15 ± 0.27	3.82 ± 0.11	3.62 ± 0.28
	**Cheese, 20 days** ^c^	8.91 ± 00.09	8.81 ± 0.16	8.92 ± 0.19	8.89 ± 0.07
	**Cheese, 40 days** ^c^	8.61 ± 0.43	8.58 ± 0.39	8.79 ± 0.16	8.78 ± 0.14
	**Cheese, 60 days** ^d^	8.11 ± 0.41	7.75 ± 0.16	8.40 ± 0.39	8.24 ± 0.32
**LAB counts (log cfu g** ^−**1**^ **)**		**0.2** ^a^	**0.5** ^a^	**0.7** ^a^	**0.9** ^a^
	**Raw milk** ^a^	2.33 ± 0.85	2.8 ± 0.16	2.26 ± 0.24	2.42 ± 0.05
	**Curd** ^b^	1.32 ± 2.28	3.06 ± 0.10	0.9 ± 1.56	0.00 ± 0.00
	**Cheese, 20 days** ^c^	8.83 ± 0.11	8.57 ± 0.38	8.63 ± 0.15	8.73 ± 0.17
	**Cheese, 40 days** ^c^	8.85 ± 0.13	8.41 ± 0.38	8.79 ± 0.16	8.66 ± 0.08
	**Cheese, 60 days** ^c^	8.77±0.10	8.19 ± 0.04	8.74 ± 0.19	8.12 ± 0.11
**CoPS counts (log cfu g** ^−**1**^ **)**		**0.2** ^a^	**0.5** ^a^	**0.7** ^b^	**0.9** ^ab^
	**Raw milk** ^a^	2.30 ± 0.30	2.18 ± 0.44	2.81 ± 0.26	2.65 ± 0.22
	**Curd** ^a^	1.00 ± 1.73	2.28 ± 1.98	2.96 ± 0.24	2.06 ± 1.79
	**Cheese, 20 days** ^b^	5.37 ± 0.22	5.21 ± 0.61	6.13 ± 0.46	5.71 ± 0.15
	**Cheese, 40 days** ^b^	4.48 ± 0.59	4.99 ± 0.90	6.25 ± 0.17	5.60 ± 0.03
	**Cheese, 60 days** ^b^	4.33 ± 0.22	4.54 ± 0.73	6.09 ± 0.21	5.50 ± 0.14

**Table 4:** Virulence factors and antibiotic resistance/sensitivity in eleven coagulase-positive staphylococci isolates obtained from the experimental cheeses. R—resistant; S—sensitive. Results of antibiotic resistance/sensitivity are given only for those antibiotics to which at least one isolate was resistant.

Isolate	DNAse	Biofilm Production (OD_570_)		Antibiotic Resistance/Sensitivity	
		24 h	48 h	Penicillin	Cefoxitin
A201	−	0.18 ± 0.07	0.20 ± 0.06	R	S
A202	−	0.16 ± 0.04	0.17 ± 0.07	R	S
A203	−	0.19 ± 0.04	0.20 ± 0.08	S	R
A204	+	0.53 ± 0.10	0.70 ± 0.14	S	S
A604	+	0.37 ± 0.16	1.17 ± 0.68	S	R
C201	+	0.18 ± 0.03	2.29 ± 0.90	S	R
C202	+	0.28 ± 0.11	1.84 ± 1.10	S	R
C203	+	0.17 ± 0.07	0.46 ± 0.30	S	R
C204	+	0.13 ± 0.08	1.23 ± 1.34	S	S
C603	+	0.15 ± 0.02	0.64 ± 0.33	S	R
C604	+	0.14 ± 0.05	1.22 ± 1.48	S	R

**Table 5:** Growth parameters of LAB (A) and staphylococcal (B) populations in whey inoculated with pure and mixed cultures of *L. lactis* L1C21M1, *S. aureus* ATCC 9144 (SA9144), and ATCC 25923 (SA25923), upon incubation at 10 °C. N_0_—initial cell numbers; λ—duration of the lag phase; µ—maximum growth rate; A—maximum cell numbers.

Inocula	N_0_	λ	µ	A	R^2^	SE of Fit
L1C21M1	6.219 ± 0.065	13.908 ± 5.162	0.033 ± 0.005	7.579 ± 0.050	0.982	0.0866
SA9144	7.079 ± 0.087	no lag	0.012 ± 0.003	8.283 ± 0.077	0.941	0.1340
L1C21M1+SA9144	7.013 ± 0.048	no lag	0.002 ± 0.001	7.565 ± 0.082	0.857	0.0948
SA25923	6.844 ± 0.012	29.437 ± 4.965	0.008 ± 0.000	8.092 ± 0.013	0.999	0.0186
L1C21M1+SA25923	6.757 ± 0.025	no lag	0.002 ± 0.000	7.222 ± 0.034	0.948	0.0479

**Table 6:** Fate of three lactococcal strains (*L. lactis* L1C21M1, L3A21M3, and L3B1M7) in whey during incubation at 4 °C for 21 days.

Lactococcal Strains	Cell Number Variation (Log Cycles)	DMFit Outputs			
		Lag Phase Length (h)	Maximum Specific Rate (h^−1^)	SE of Fit	R^2^
L1C21M1	+1.460	14.077 ± 5.486	0.030 ± 0.005	0.081	0.985
L3A21M1	−0.619	--	−0.002 ± 0.000	0.051	0.982
L3B1M7	+3.050	0	0.027 ± 0.005	0.051	0.964

## Data Availability

The original contributions presented in this study are included in the article. Further inquiries can be directed to the corresponding author.
